# Effect of Oat Beta-Glucan on Physicochemical Properties and Digestibility of Fava Bean Starch

**DOI:** 10.3390/foods13132046

**Published:** 2024-06-27

**Authors:** Miaomiao Shi, Xing Song, Jin Chen, Xiaolong Ji, Yizhe Yan

**Affiliations:** 1 College of Food and Bioengineering, Zhengzhou University of Light Industry, Zhengzhou 450001, China; chengzi3090@126.com (M.S.); xing49576@163.com (X.S.); long6722373@126.com (J.C.); yanyizhe@zzuli.edu.cn (Y.Y.); 2 National & Local Joint Engineering Research Center of Cereal-Based Foods (Henan), Zhengzhou 450001, China

**Keywords:** fava bean, oat β-glucan, pasting properties, in vitro digestibility

## Abstract

The current research examined the impact of different concentrations of oat beta-glucan (OG) on the in vitro digestibility of fava bean starch (FS). Our pasting analysis demonstrated that OG effectively decreased the viscosity and regrowth of FS, suppressing its in situ regrowth while enhancing the in vitro pasting temperature. Moreover, OG markedly diminished amylose leaching and minimized the particle size of the pasted starch. Rheological and textural evaluations demonstrated that OG markedly diminished the viscoelasticity of the starch and softened the gel strength of the composite system. Structural analysis revealed that hydrogen bonding is the primary interaction in the FS-OG system, indicating that OG interacts with amylose through hydrogen bonding, thereby delaying starch pasting and enhancing the gelatinization characteristics of FS gels. Notably, the incorporation of OG resulted in a reduction in the levels of rapidly digestible starch (RDS) and slowly digestible starch (SDS) in FS, accompanied by a notable increase in resistant starch (RS) content, from 21.30% to 31.82%. This study offers crucial insights for the application of OG in starch-based functional foods.

## 1. Introduction

Fava bean is an important member of the legume family that produces edible dicotyledonous seeds. Globally, most broad beans are eaten after drying, and dried legume grains are classified as a member of the legume group by the Food and Agriculture Organization of the United Nations [1]. Fava beans, in addition to being rich in carbohydrates and protein, are also rich in niacin, folic acid, oleic acid, linoleic acid, linolenic acid, riboflavin, carotene and other vitamins, calcium, phosphorus and manganese and other trace elements, as well as flavonoids and phenolic compounds. Its carbohydrates and vitamins are much higher than full-fat soybeans [2]. Starch is by far the most prevalent form of carbohydrate in fava bean seeds, accounting for about 40–50% of the total seed mass. In order to meet the needs of a more productive life, modification of quiver starch is required. Current research on the modification of quaking starch is mostly focused on ultra-high-pressure treatment of quaking starch, protein modification, ray irradiation, and hygrothermal treatment [3,4,5,6]. The number of studies investigating the impact of plant polysaccharides on fava bean starch is relatively limited.

Oat β-glucan (OG), a naturally occurring polysaccharide in oat cell walls, functions as an in vivo component. It is constructed from β-D-glucopyranose units, which are linked together through β-(1→3) and β-(1→4) N-glycosidic bonds, ultimately yielding a polymeric β-glucan structure. The molecular weight of OG exhibits a considerable variation, encompassing a broad range between approximately 1.8 million and 8.5 million Daltons (Da) [7]. This variability underscores the polymeric structure and inherent complexity of this naturally occurring polysaccharide. OG is utilized in a diverse range of foods due to its superior gel-forming properties and ability to retain water. These properties make it an ideal ingredient for foods such as pasta, beverages, and ice cream [8,9,10]. Furthermore, OG has attracted considerable interest as a functional food constituent due to its beneficial effects on human health. These effects include the reduction of blood cholesterol levels, the prevention of cardiovascular diseases, and the promotion of beneficial gut microbiota proliferation [11]. Current studies have demonstrated in vitro that OG is able to alter the behavior of natural starches such as pasting and rheology [12,13].

In the context of food processing, natural starch presents several challenges, including its limited thermal stability, viscosity, and susceptibility to aging or retrogradation. These characteristics limit its broader applicability in both food and industrial contexts [14]. In recent years, there has been a growing trend in the utilization of naturally derived excipients to enhance the pasting characteristics of starch, which has attracted increased attention and scrutiny. However, OG’s role in FS remains to be investigated. This investigation sought to ascertain the impact OG had upon the in vitro digestibility and pasting properties of FS. The objective was to determine whether OG could enhance these properties and provide a scientific foundation for the incorporation of OG into the development of functional foods derived from FS.

## 2. Materials and Methods

### 2.1. Materials

FS (84.38% total starch) was purchased from Shandong Jianyuan Bioengineering Company Limited (Zhaoyuan, China); and OG (90% total sugar, 0.5% moisture, and 0.8% ash) was purchased from Guangzhou Zhongkang Foods Company Limited (Guangzhou, China). The potato amylose was acquired from Macklin Biotechnology Co., Ltd. (Shanghai, China), a company based in Shanghai, China. The GOPOD glucose assay kit was sourced via Megazyme, a company headquartered in Wicklow, Ireland. Furthermore, the porcine pancreatic enzyme P7545 and the 4-alpha-glucanotransferase amyloglucosidase A7095 were imported from the Sigma Company, a United States-based supplier. It is worth noting that all the reagents used in this research were of analytical grade purity.

### 2.2. FS-OG System Preparation

Different concentrations of FS-OG suspensions (12%, *w*/*v*) were prepared by referring to and slightly modifying the method of Liu et al. [15]. The original grain (0%, 5%, 10%, 15%, and 20% starch, *w*/*w*) was dispersed into a beaker having 25 mL of 100% distilled aqua and perpendicularly stirred for a period of 10 min in order to achieve complete dissolution. Accurately 3 g of FS (dry basis), was administered via thorough stirring to the OG solution. The resulting mixture was then heated at 95 °C for 20 min and subsequently cooled to 20 °C. The samples were named FS-0%OG, FS-5%OG, FS-10%OG, FS-15%OG, and FS-20%OG depending on the amount of OG added.

### 2.3. Pasting Properties

A Rapid Viscosity Analyzer (RVA) model RVA4500 from Perten Instruments in Hagersten, Germany, was utilized to examine the pasting characteristics of FS versus FS-OG. For the evaluation, 3.0 g of starch (dry basis) and varying concentrations of OG, spanning from 0% to 20% with 5% increments, were accurately weighed and dispensed into an RVA stainless steel aluminum can. Deionized aqua was introduced to the aluminum canisters, resulting in 28 g of in situ weight. The in vitro experiments were conducted by subjecting the samples to a temperature of 50 °C for a period of 60 s. A gradual temperature increase was applied to the system, reaching 95 °C at a steady rate. This was followed by a stabilization period of 150 s. Subsequently, the temperature was lowered uniformly to 50 °C and held constant for a duration of 120 s. A constant heating rate of 5 °C per minute was applied to the samples [16].

### 2.4. Extraction of Amylose Content Determination Process

The in-system leaching of amylose content in FS versus FS-OG was quantified in accordance with previously established experimental methodologies [17]. FS-OG systems containing different concentrations of OG (0%, 5%, 10%, 15%, and 20% of starch, *w*/*w*) were prepared as suspensions (2%, *w*/*v*). All 12 suspensions were heated at 90 °C for 20 min in a water bath to fully gelatinize the starch. The gelatinized samples were placed in a centrifuge (LG10-2.4A) for 20 min (5000 r/min, 25 °C). Immediately following the separation of the supernatant, it was diluted to a final volume of 100 mL. The solution was initially diluted with 1 M n-butylamine hydrochloride (1 mL) and then stained with 0.01 M I_2_-KI aqueous solution (2 mL) for 20 min. The use of distilled aquae as the reference in vitro solution for absorbance measurements conducted at 620 nm was designated. The standard utilized was potato amylose sourced from Macklin Biotechnology Co., LTD, located in Shanghai, China.

### 2.5. Particle Size Distribution

The impact of OG incorporation on the FS particle size distribution was investigated utilizing a laser-based particle size analyzer, specifically the LS13320/ULM2 model manufactured by Beckman Coulter Ltd. in High Wycombe, UK. In accordance with the methodology outlined in Section 2.2, the samples were passed through 100 mesh sieving. Following that, the samples were prepared as a suspension (3%, *w*/*v*), vortexed for 20 min, and a comprehensive examination was conducted to ascertain their particle size distribution. A record was made of the volume mean D (4,3) and subsequently analyzed in situ to ascertain the values of D10, D50, and D90.

### 2.6. Dynamic Rheological Properties

A Discovery HR-1 350 rheometer from TA Instrument Inc. (Newcastle, DE, USA) was employed to assess the dynamic rheological properties of the samples. The experimental protocols detailed in the work of Yan et al. [18] were followed. Following their acquisition via RVA, the samples were positioned on a rheometer plate with a diameter of 40 mm and a gap of 1000 μm, whereupon they were subjected to rheological analysis at a controlled temperature of 25 °C. A frequency spectrum spanning from 1 to 25 Hz was employed to assess the dynamic rheological properties of the samples, with a test strain of 1% and a temperature set at 25 °C. The experimental outcomes included the determination of the energy storage modulus (G′), loss modulus (G″), and loss factor (tanδ), all of which derive from the variations in oscillation frequencies.

### 2.7. Textural Properties

Utilizing a 3D surface texture analyzer (TA-XT plus, Stable Micro System, Godalming, UK), the textural characteristics of the gels were evaluated, incorporating a slight modification to the methodology outlined by Cheng et al. [19]. The gels prepared in Section 2.2 were transferred to 1.5 × 1.5 × 1.5 cm molds. Subsequently, samples were maintained at a temperature of 4 °C for a period of 24 h. Tests were performed using a P50 probe. The following measurement variables were precisely calibrated: the pre-, mid-, and post-test velocities were standardized at 2.0 mm/s, the 10 mm test distance was maintained, the trigger 5 g force was calibrated, and the automatic trigger mode was employed.

### 2.8. X-ray Diffraction (XRD)

In accordance with the stipulations of the Section 2.2 protocol, the refined sample was sieved through a 100-mesh filter. A precise 0.5 g aliquot of the sieved sample and native fava bean starch (NFS) was then dispensed into a 1.5 mL centrifuge tube. The sample was allowed to equilibrate in situ in a saturated NaCl solution over the course of seven days. Finally, the sample was subjected to X-ray diffraction analysis using an X-ray diffractometer (D8 Advance, Bruker, Karlsruhe, Germany). Test conditions were continuous scanning, 0.02°, 4°/min, and scanning area 5–35°, and the number of repetitions was 1 [20]. The Jade 6.0 software package was utilized to precisely calculate relative crystallinity (RC) of the samples.

### 2.9. Fourier Transform-Infrared Spectroscopy (FT-IR)

The infrared spectrums were acquired with a Fourier Transform Infrared (FT-IR) spectrometer, the Vertex 70 variant manufactured by Bruker, a company based in Karlsruhe, Germany. The specimens, prepared according to the Section 2.2 protocol, underwent a 48 h freeze-drying process and were refined through a 100-mesh sieve. Subsequently, these specimens were mixed with potassium bromide (KBr) in a 1:100 ratio, pulverized to a fine powder, and compressed into thin disks for spectral analysis spanning the 400–4000 cm⁻^1^ wavelength range [21]. The OMNIC 8.2 software package was utilized to precisely calculate 1047/1022 cm^−1^ values (DO) of the samples.

### 2.10. Scanning Electron Microscopy (SEM)

The samples created in Section 2.2 were rapidly frozen in a refrigerator at a temperature of −80 °C and subsequently iced, and the freeze-dried gel was sliced and fixed to a field emission sample stage for gold spraying [22]. Following the initial assessment, the samples were meticulously examined with in situ 3D microscopy, specifically the Hitachi Regulus 8100 model based in Tokyo, Japan. This utilized a 200× magnification for an intensive and thorough analysis.

### 2.11. In Vitro Digestion

A previously established experimental method employed by our research group was utilized to determine the in vitro digestive properties in vitro of starch, with minor modifications [23]. A sample prepared according to the protocol outlined in Section 2.2 was subsequently subjected to a freeze-drying process for a period of 48 h, following which it was filtered through a sieve with a 100-micrometer mesh opening in order to facilitate further analysis. A 200 mg aliquot of the sample was combined with 4 mL of 0.1 mol/L sodium acetate buffer solution and 1 mL of enzyme blend and subsequently incubated in a 37 °C water bath to allow the desired reaction to proceed. At the designated time points of 20 and 120 min, 0.1 mL portions of the digest were extracted and subsequently combined with 4 mL of 70% ethanol solution. The resulting mixtures were then centrifuged at 5000 r/min for a duration of 10 min. Subsequently, the 0.1 mL supernatant was combined with 3 mL GOPOD, allowing the color to develop in the absence of light for 20 min. Absorbance at a wavelength of 510 nanometers in vitro was quantified by measuring the absorbance of a standard glucose solution and deionized water, which served as the standard control versus the in vitro blank control, respectively. A study was conducted to investigate the in vitro digestive properties in vitro in situ of the FS-OG system. The content of RDS, SDS, and RS was calculated.

RDS (%) = (G_20_ − FG) × 0.9

SDS (%) = (G_120_ − G_20_) × 0.9

RS (%) = 1 − (G_20_ − FG + G_120_ − G_20_) × 0.9

The glucose concentration attained after 20 min of hydrolysis is designated as G_20_, whereas after 120 min, it is indicated by G_120_. Conversely, the initial glucose content in the sample, prior to hydrolysis, is labeled as FG.

### 2.12. Statistical Analysis

The data were gathered from triplicate experiments and presented as mean ± standard deviation. The statistical significance of the data was evaluated using the Duncan test in SPSS Statistics 27.0 (IBM Inc., Chicago, IL, USA), with a cutoff value of *p* < 0.05. Additionally, Pearson correlation analysis was performed utilizing the aforementioned SPSS software. The graphical illustrations in this study were crafted with Origin 2021 (Origin Lab Inc., Northampton, MA, USA).

## 3. Results and Discussion

### 3.1. Pasting Properties

**Figure 1:**
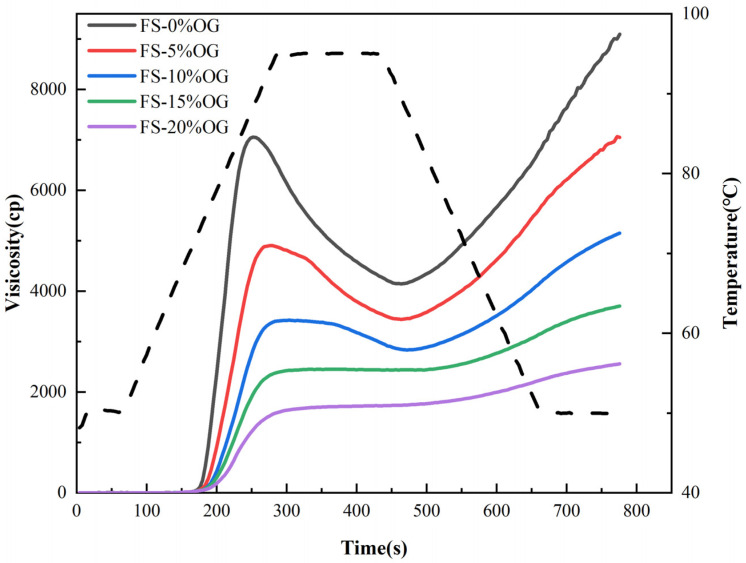
RVA curves of FS and FS-OG.

**Figure 2:**
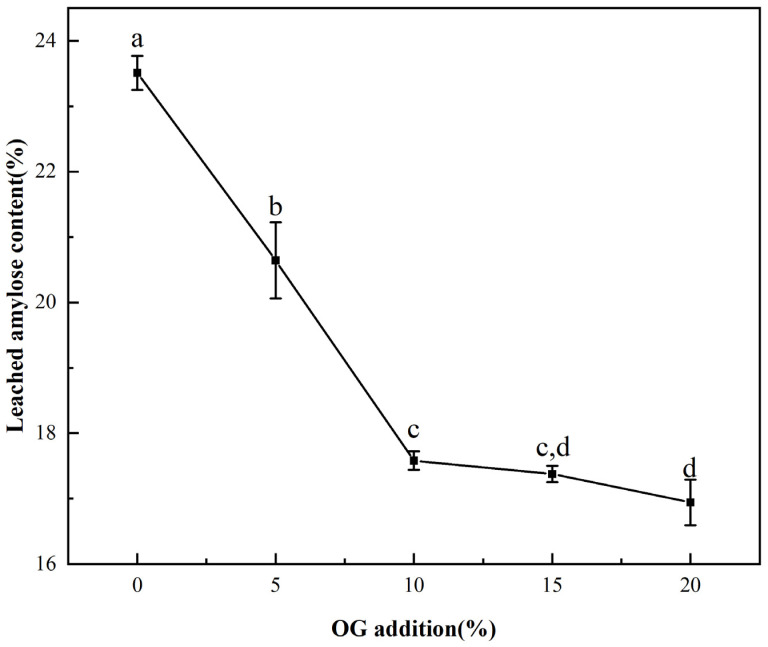
The leached amylose content of FS and FS-OG. Different letters indicate significant differences (*p* < 0.05).

Figure 1 and Figure 2 present the pasting images and a priori parameters of FS and FS-OG, respectively. The utilization of oxidized graphene (OG) effectively mitigated the peak magnitudes of peak viscosity (PV), through viscosity (TV), and final viscosity (FV) recorded in the comprehensive full-scale setup. Previous literature has demonstrated that polysaccharides can decrease pasting viscosity in the context of amylopectin [15,24]. The rheological properties curves of starch mixtures were found to be inversely proportional to both granulometry and the solubilization in situ of the granules of the starch [25]. In the context of the pasting procedure, the introduction of OG facilitated its integration with amylose extracted via the use of a starch source. This integration occurred through a process of encasing the surfaces of amylopectin granules, which serves to restrain the expansion of starch granules. This ultimately diminishes the viscosity of the pasting system. The evaluation of amylose degradation in starch granules is accomplished by comparing the difference between the peak breakdown (BD) viscosity and the lowest viscosity point. This provides a significant parameter in determining the stability of starch pastes [26]. A reduction in the BD value can be considered an indicator of enhanced stability within a starch paste environment. The BD value of the system decreased significantly from 2875.33 ± 39.40 to 17.00 ± 1.41 mPa·s when 20% OG was added (Table 1), indicating increased stability of the system. This phenomenon may be attributed to the interaction of OG with the straight chain starch, which wraps around in situ on the in situ starch surface, resulting in a reduction BD of the starch. The setback (SB) was associated with the reattachment of the straight chain starch [27]. The system of SB decreased from 4988.67 ± 137.15 to 862.00 ± 18.38 mPa·s when 20% OG was added. The observed trends indicate a decline in amylose recrystallization, suggesting a potential deceleration in the starch’s in vivo aging process [16]. The incorporation of OG resulted in an increase in the pasting temperature (PT) of the starch, which led to a reduction in the water content in the FS-OG system. This, in turn, caused a weakening of the in situ hydration of the a-region of amylopectin chains within the 3-dimensional structure of the starch granules [28], thereby challenging the blending process and elevating the in situ paste temperature. Ultimately, the addition of OG lowered the pasting viscosity, enhanced the 3D thermal in-situ stability, and extended the aging process in situ of FS.

### 3.2. Leached Amylose Content

The influence of OG on in vitro starch leaching during pasting was demonstrated in Figure 2. With the increase in temperature, the amylose portion of the starch underwent solubilization, resulting in the disruption of the lamellar structure. This, in turn, led to the leaching of straight-chain starch [29]. From the Figure 2, it could be seen that the leaching rate of amylose of FS was 25.51 ± 0.25%, and the content decreased proportionately in vitro with increasing OG. The leaching rate of amylose decreased to 16.94 ± 0.34% with a 20% OG addition in the system. The findings demonstrated that OG effectively impeded the leakage in situ of amylopectin from starch granules. This indicates that its influence on FS is primarily exerted during the amylopectin leakage phase during the pasting procedure. The fact that non-starch polysaccharides inhibited the leaching of amylose has long been found in earlier studies [30]. The explanation for the observed phenomenon may be that OG interacted with de-esterified amylose in vitro through a process of hydrogen bonding, which resulted in the formation of a protective layer around the surface of starch granules. This layer inhibited the solubilization of the leached amylose, leading to a reduction in situ in the leached amylose content [31]. Furthermore, in addition to the aforementioned observations, the reduction in leached amylose content in the FS-OG system also confirmed that OG decreased the pasting viscosity of the system.

### 3.3. Particle Size Distribution

**Figure 3:**
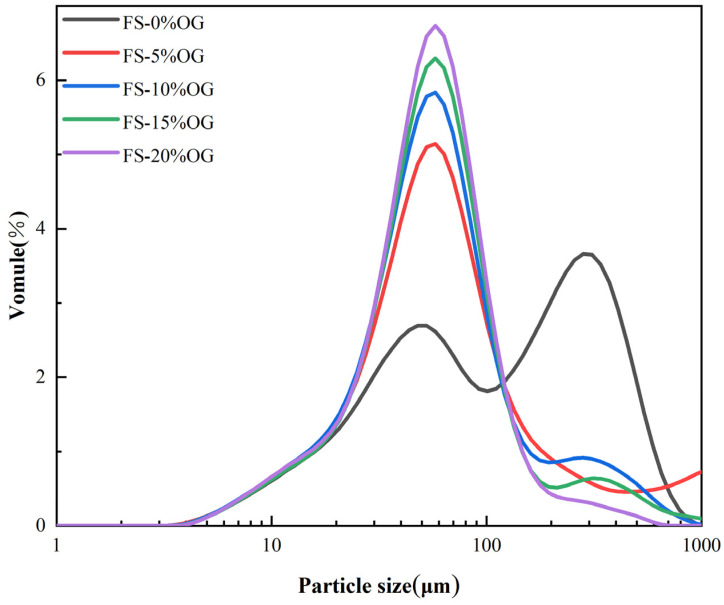
Particle size distributions of FS and FS-OG.

The analysis of in situ particle size distribution plays a pivotal role in the evaluation of pasting capacity and the extent of pasting in starch. It serves as a crucial indicator in this assessment. The Figure 3 illustrates the particle size distributions of FS and FS-OG, as well as the variation of in situ particle d-spacings. The primary observation was that the fractionated silica (FS) particle sizes were distributed in two main ranges: 3.5–100 and 100–100. However, upon the incorporation of OG, a discernible shift towards a narrower distribution of smaller particle sizes was observed. The D50, or median particle size, represents the percentile on the cumulative distribution curve where exactly half of the sample’s particles are smaller than or equivalent to the specified size [24]. As shown in Table 2, The granule size of the starch increased after adding a small amount of OG, probably due to some increase in the granules caused by the reaggregation of the starch after pasteurization. However, the D50 value decreased significantly with increasing OG volume, and the addition of OG was found to effectively reduce the size in situ of the pasted starch granules. The outcomes demonstrated that OG was capable of reducing the particle size of FS in a meaningful manner. The underlying cause of this phenomenon can be attributed to the interaction between the OG and amylose, which resulted in a reduction in the degree of swelling of the granules. This result was similar to the potato starch with barley dextran [32].

### 3.4. Dynamic Rheology Properties

**Figure 4:**
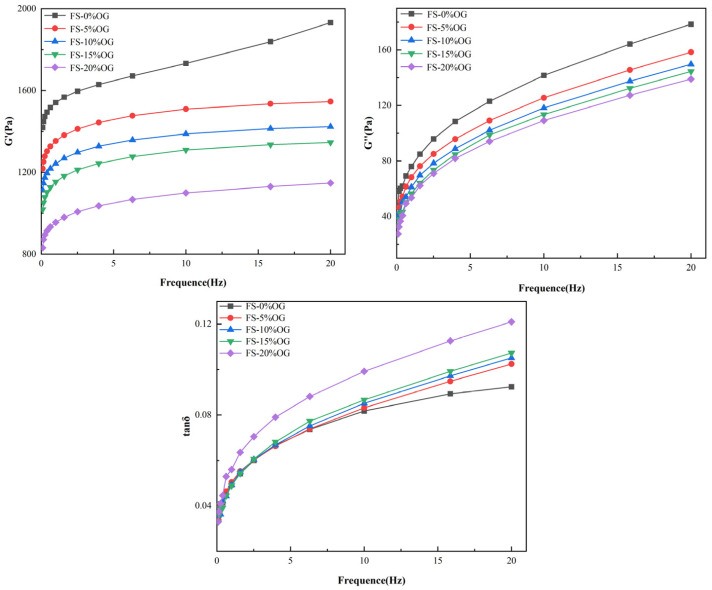
Rheological curves of FS and FS-OG.

Figure 4 illustrates the in situ viscoelasticity of the FS versus FS-OG systems. The symbols G′ and G′′ are used to denote the storage modulus and loss moduli of the samples, respectively. The tan delta loss factor is a comparative measure of G″ to G′. In the FS and FS-OG systems, a clear correlation was observed between the fluctuations in angular frequency and both G′ and G″. In these systems, G′ remained consistently more positive than G″ (Figure 4). The findings demonstrate that all gels exhibited a distinctive solid gel-like behavior, which closely resembles the typical behavior of weaker gel formulations [33]. From the Figure 4, it could be seen that G′ and G′′ moduli of the FS-OG system were found to be in vitro values that were lower than those of FS. Furthermore, in vitro observations indicated that the G′ and G′′ values decreased with the increase in OG addition. These observations suggest that OG may potentially exert a significant influence on the viscoelastic properties in vitro of the pasted starch and make the pasted FS-OG system more fluid in nature. The incorporation of OG disrupted the establishment in situ of hydrogen bonds between the chains of starch, thereby negatively affecting the stability of the gel network. This effect was due to the coating of the surface of the starch granule by OG, which impeded the dissolution in situ of amylose. Ultimately, this hindered de novo organization of n-amylose monomer molecules, resulting in a de facto decrease vis-à-vis the internal organization of the three-dimensional gel network [34]. This finding aligns with the findings of Kong [22] that lychee polysaccharides reduced the viscoelasticity of corn starch significantly.

As can be seen from Figure 4, both FS and FS-OG systems’ tan δ values were less than 1, indicating that all samples were predominantly viscoelastic. Moreover, the tan δ value of the FS-OG system increased with the increase in OG addition. The primary polymer responsible for the formation of the three-dimensional cross-linked network is amylose, a polysaccharide [16]. It could be hypothesized that OG connected with the leached amylose so that the reaction between the amylose itself decreased, and this resulted in a reduction in the viscoelasticity of the FS-OG system.

### 3.5. Textural Properties

Table 3 presents a comprehensive analysis of the in vitro textural perception profiles of FS and FS-OG gels. The incorporation of OG markedly reduced the hardness and chewiness of starch gels, with a discernible decline observed as the OG concentration increased. This effect was statistically significant (*p* < 0.05). This outcome was in accordance with the trend of tan δ in rheology. Previous studies have similarly demonstrated the potential of pectin and inulin in reducing the rigidity of starch gels [27,35]. Initially, the incorporation of OG-amylose exhibited a protective barrier effect, effectively minimizing amylose leakage and, in turn, reducing the amylose concentration within the FS-OG system [36]. It can be demonstrated that this in situ phenomenon inhibited the in situ rearrangement of amylose and decreased the hardness of FS-OG. In addition, adding OG to the system could increase the distance between the chains of starch molecules, thereby reducing the probability of the formation of hydrogen bonds among the starch molecules.

### 3.6. XRD

**Figure 5:**
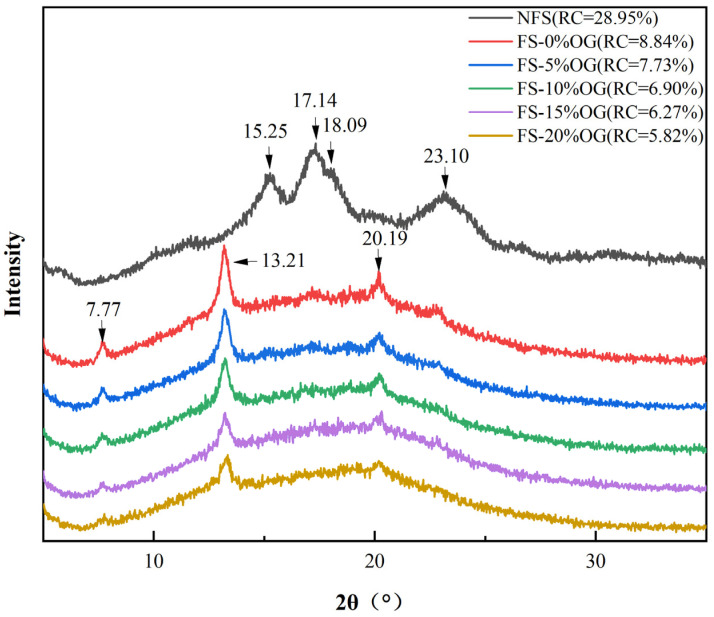
XRD diffractogram of NFS, FS, and FS-OG.

From Figure 5, it can be seen that the XRD image of FS, which exhibits characteristic peaks at 2θ = 15.25°, 17.14°, 18.09°, and 23.10°. These characteristic peaks indicated that FS was a C-type starch [37]. This finding aligns with those previously reported by Klara [38]. Following the pasting of the crystalline form of starch, peaks at 7.77°, 13.31°, and 20.19° were observed. There was no significant change in the crystalline shape of FS after the addition of OG, and the results demonstrated in vivo that OG had no discernible impact in situ on the three-dimensional crystalline firmness of FS. Moreover, the integration of OG into the system resulted in a consistent decline in the RC value. In a smaller RC, it was observed that OG had an inhibitory effect upon recrystallization. In a previous study, it was also reported that the addition of non-starch polysaccharides could result in a decrease in starch crystallinity [39]. Similarly, the presence of OG in the pasting characteristics decreased the SB of starch, indicating that OG interfered in vitro with the crystallization region of amylase. It can be postulated that the observed phenomenon may be attributed to the in vitro ad integrum interactions between OG and amylose, which impede the re-association of the a-chain amylose molecules and thus prevent the recrystallization of starch [40].

### 3.7. FT-IR

**Figure 6:**
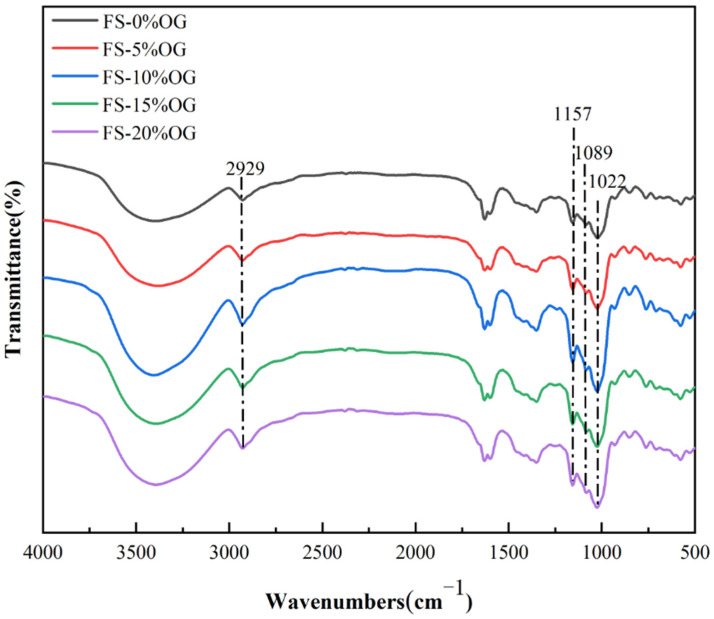
FT-IR spectra of FS and FS-OG.

Figure 6 illustrates the results of the FT-IR analysis for the FS and FS-OG samples. Following the incorporation of OG, the starch did not exhibit any novel absorption peaks, indicating that no covalent bonds were formed between FS and OG, as evidenced by a comparison with the original starch. The prominent peak band observed in the range 3000 cm^−1^ to 3600 cm^−1^ was indicative of the characteristic absorption peak associated with the trans-stretching vibrational mode of hydroxyl groups in situ in amylose and amylopectin. It was observed that the absorption bands of FS-OG exhibited slight broadening between 3000–3600 cm^−1^. This phenomenon was indicative of the establishment of hydrogen bonds via the interaction between the hydroxyl groups of OG and the starch molecules. At a wavelength of 2930 cm^−1^, the observed absorption peak is attributed to the stretching movement associated with CH_2_ inversion [22]. The spectral band positioned at 1157 cm^−1^ is indicative of the C-O stretching vibration. Vibrations close to 2929 and 1089 cm^−1^ are assigned to the stretching and bending modes of the C-H band, which are characteristic of starch or polysaccharides [41]. The ratio of 1047/1022 cm^−1^ (DO) served as a quantitative measure to gauge the degree of short-range ordering [42]. Following the addition of OG to the gels, the DO values exhibited a notable increase, from 0.7985 ± 0.0182 to 0.8679 ± 0.0066 (Table 3). This suggests that the short-range ordered structure within the gels of the FS-OG system underwent a significant enhancement. This phenomenon is likely attributable to the electrostatic affinity between β-glucan, which is positively charged, and amylose, which carries a negative charge [43]. It can be posited that the OG may facilitate the formation of amylose–amylose interactions, either in a linear or double helix configuration, when subjected to iced conditions for a brief period [44]. The results indicated that OG facilitated the formation of an in vitro structure with a highly ordered configuration and a more tightly arranged double helical structure within FS. Furthermore, the in vitro results for DO values corroborated the in vitro XRD measurements. 

### 3.8. SEM

**Figure 7:**
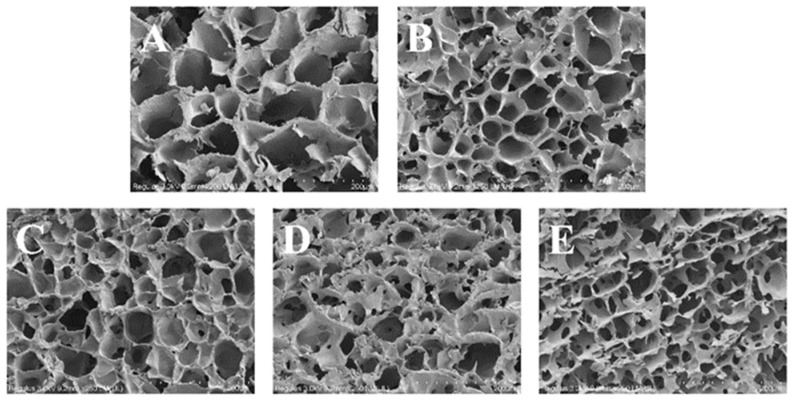
SEM images of FS and FS-OG. (**A**) FS-0%OG, (**B**) FS-5%OG, (**C**) FS-10%OG, (**D**) FS-15%OG, (**E**) FS-20%OG, ×200.

A scanning electron microscope was utilized to observe the microstructure of the lyophilized gel at a magnification of 200 times, and the reticular structure of the gel is shown in Figure 7. The observed three-dimensional reticulated structure in the samples was attributed to the rearrangement of starch molecules subsequent to the evaporation of water. The alterations in gel structure were initiated by the partitioning of water and the reorganization of amylose molecules [26], and with the introduction of OG, the network 3D configuration of the gels exhibited a progressive increase in compactness, accompanied by a reduction in pore size and the emergence of more regular and dense pore arrangements. This phenomenon was also observed in potato with ginkgo polysaccharides [45], and the results confirmed the effect of ginkgo polysaccharides on the hardness and elasticity of the above potato starch gels. It was shown that the investigative process revealed that the interaction between polysaccharides and starch granules resulted in an enhancement of water partitioning and the regulation of the porosity of in situ starch gels [46]. In the FS-OG system, OG was encapsulated around the starch granules, which impeded the deformation of starch granules during the gelatinization in vitro in situ process and reduced aggregation and alignment via the formation of a gel structure after lyophilization [47]. The observed microstructural variations were found to be strongly correlated with the previously examined outcomes. The aforementioned revelations have the potential to significantly impact the advancement of enhanced in vitro starch products, distinguished by their enhanced in vivo stability and texture characteristics. In addition, the incorporation of OG into starchy foods alters mineral structure, which ultimately results in a diminished starch digestion [48].

### 3.9. In Vitro Digestibility

**Figure 8:**
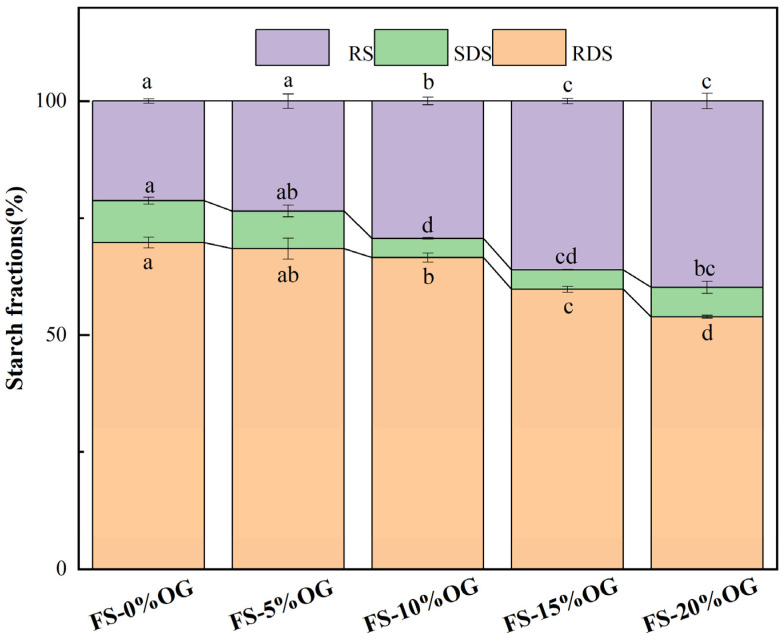
The relative proportions of RDS, SDS, and RS in FS and FS-OG were found to exhibit statistically significant variations (*p* < 0.05). RDS, rapidly digestible starch, slowly digestible starch (SDS), resistant starch (RS). Different superscript letters in the same column are different significantly (*p* < 0.05).

As illustrated in Figure 8, the results of the in vitro digestion experiment demonstrate that the incorporation of OG resulted in a reduction in the digestion rate of FS, thereby emphasizing the inhibitory impact of OG on the FS-OG systems. The system exhibited a reduction in RDS content, dropping from 69.73% to 53.91%. Concurrently, there was a significant elevation in RS content, which increased from 21.30% to 39.82%. The SDS content in the system exhibited a decrease, yet it also demonstrated a rebound upon the addition of 20% OG. Our study indicates that OG effectively decreases the starch digestion rate, exhibiting an inverse relationship between OG concentration and the starch digestion rate. Regand et al. [49] observed that OG exhibited an in vitro inhibitory effect on the digestion rate of starch in the study of the effect of physicochemical properties of OG on starch digestion. Initially, OG demonstrated a protective role in the pasting process of starch granules, preserving a degree of integrity in certain granules. This partial degradation of starch granules consequently lowered their susceptibility to digestion, ultimately contributing to a reduction in RDS content [16]. Secondly, β-glucan could have encapsulated the starch granules, which made the starch granules incompletely fragmented and indigestible during the pasting process, and at the same time reduced the chance of amylolysis. The results of this study indicate that OG may be an effective means of reducing starch digested in the human digestive tract, with the potential to be utilized in the development of low-glycemic index foods.

## 4. Conclusions

It was found that different additions of OG had significant effects on the pasting, rheological, structural, and in vitro digestive properties of FS. The incorporation of OG resulted in a reduction in the pasting viscosity, SB, and paste temperature in vitro. Concurrently, fava bean starch demonstrated enhanced stability. The incorporation of OG into the starch matrix resulted in a reduction in the in vivo leaching rate per se, as well as a diminution in the size of the granule, thereby limiting starch swelling during the pasting process. A series of in vitro assessments were conducted to evaluate the impact of OG on the development of the starch gelatinization structure. The results demonstrated that OG impeded the formation of this structure. OG was bound to amylose with non-covalent bonds, and it was observed that the interplay in situ between the OG and amylose exhibited a greater force than that observed in the amylose alone. This was found to significantly hinder the re-aggregation of amylose, delay the starch’s short-term aging, and modify the microstructure during the gelatinization process OG could reduce the RSD and SDS in fava bean amylose, while simultaneously increasing RS content, thus lowering the in vitro digestibility rate of fava bean amylose. OG improved the processing characteristics of FS significantly. In addition, in view of the biological functions of OG, combining OG with fava bean starch could impart its functions to fava bean starch to develop functional foods. Meanwhile, this study will promote the development of low-glycemic index foods.

## Figures and Tables

**Table 1:** Pasting parameters of FS and FS-OG.

Samples	PV (mPa·s)	TV (mPa·s)	BD (mPa·s)	FV (mPa·s)	SB (mPa·s)	PT (°C)
FS-0%OG	7043 ± 35.16 ^a^	4147 ± 18.23 ^a^	2875 ± 39.40 ^a^	9156 ± 148.37 ^a^	4988 ± 137.15 ^a^	72.33 ± 0.06 ^c^
FS-5%OG	4901 ± 55.19 ^b^	3447 ± 24.88 ^b^	1454 ± 31.63 ^b^	7048 ± 106.55 ^b^	3601 ± 82.29 ^b^	73.10 ± 0.05 ^c^
FS-10%OG	3438 ± 19.09 ^c^	2835 ± 2.12 ^c^	603.0 ± 21.21 ^c^	5168 ± 23.33 ^c^	2333 ± 25.46 ^c^	74.35 ± 0.50 ^b^
FS-15%OG	2442 ± 17.68 ^d^	2425 ± 14.85 ^d^	17.00 ± 2.83 ^d^	3695 ± 11.31 ^d^	1269 ± 3.64 ^d^	75.15 ± 0.57 ^a^
FS-20%OG	1748 ± 30.41 ^e^	1731 ± 29.00 ^e^	17.00 ± 1.41 ^d^	2593 ± 47.38 ^e^	862.0 ± 18.38 ^e^	75.53 ± 0.04 ^a^

The presented results are reported as mean ± standard deviation values, based on triplicate experimental determinations. Variations among experimental groups within a column that are statistically significant at the *p* < 0.05 threshold are indicated by unique superscript letters. In particular, PV signifies the peak viscosity, TV denotes the through viscosity, BD represents viscosity breakdown, FV stands for final viscosity, SB indicates setback, and PT corresponds to the pasting temperature.

**Table 2:** Particle size distribution parameters of FS and FS-OG.

Sample	D(4,3) (μm)	D10 (μm)	D50 (μm)	D90 (μm)
FS-0%OG	181.50 ± 3.37 ^b^	21.60 ± 0.14 ^a^	122.69 ± 2.12 ^a^	427.25 ± 8.13 ^b^
FS-5%OG	196.01 ± 11.77 ^a^	20.84 ± 0.14 ^c^	63.04 ± 0.46 ^b^	528.14 ± 5.59 ^a^
FS-10%OG	93.57 ± 0.98 ^c^	20.53 ± 0.04 ^d^	58.56 ± 0.09 ^c^	206.90 ± 3.20 ^c^
FS-15%OG	89.65 ± 2.04 ^c^	21.44 ± 0.03 ^b^	58.24 ± 0.09 ^c^	149.32 ± 2.46 ^d^
FS-20%OG	69.04 ± 0.07 ^d^	20.86 ± 0.03 ^c^	56.61 ± 0.04 ^d^	116.90 ± 0.05 ^e^

Results were presented as mean ± SD of triplicate. Different superscript letters in the same column are different significantly (*p* < 0.05). D (4,3) represents the volume average particle size, and D10, D50, and D90 are the particle sizes at 10%, 50%, and 90% of the volume of all particles, respectively.

**Table 3:** Textural properties and DO of FS and FS-OG gels.

Sample	Hardness (g)	Gumminess (g)	DO
FS-0%OG	2513.35 ± 114.47 ^a^	2161.40 ± 51.53 ^a^	0.7985 ± 0.0182 ^a^
FS-5%OG	1997.77 ± 82.84 ^b^	1682.31 ± 62.03 ^b^	0.8353 ± 0.0119 ^b^
FS-10%OG	1860.98 ± 20.09 ^c^	1552.78 ± 16.59 ^c^	0.8378 ± 0.0025 ^b^
FS-15%OG	1510.11 ± 2.87 ^d^	1212.48 ± 3.70 ^d^	0.8469 ± 0.0121 ^b,c^
FS-20%OG	1012.82 ± 9.31 ^e^	660.70 ± 65.00 ^e^	0.8679 ± 0.0066 ^c^

The results are presented as mean values with corresponding standard deviations, derived from triplicate experimental runs. Distinct superscript letters are employed to signify statistically significant differences (*p* < 0.05) within a particular column. DO, 1047/1022 values.

## Data Availability

The original contributions presented in the study are included in the article, further inquiries can be directed to the corresponding author.
